# Super-Resolution Enhancement Method Based on Generative Adversarial Network for Integral Imaging Microscopy

**DOI:** 10.3390/s21062164

**Published:** 2021-03-19

**Authors:** Md. Shahinur Alam, Ki-Chul Kwon, Munkh-Uchral Erdenebat, Mohammed Y. Abbass, Md. Ashraful Alam, Nam Kim

**Affiliations:** 1 Department of Computer and Communication Engineering, Chungbuk National University, Cheongju, Chungbuk 28644, Korea; shahinur@chungbuk.ac.kr (M.S.A.); kwon@osp.chungbuk.ac.kr (K.-C.K.); uchka@osp.chungbuk.ac.kr (M.-U.E.); myehiaa@chungbuk.ac.kr (M.Y.A.); 2 Department of Computer Science and Engineering, BRAC University, Dhaka 1212, Bangladesh; ashraful.alam@bracu.ac.bd

**Keywords:** deep learning, generative adversarial network, integral imaging microscopy, machine intelligence, microscopy

## Abstract

The integral imaging microscopy system provides a three-dimensional visualization of a microscopic object. However, it has a low-resolution problem due to the fundamental limitation of the F-number (the aperture stops) by using micro lens array (MLA) and a poor illumination environment. In this paper, a generative adversarial network (GAN)-based super-resolution algorithm is proposed to enhance the resolution where the directional view image is directly fed as input. In a GAN network, the generator regresses the high-resolution output from the low-resolution input image, whereas the discriminator distinguishes between the original and generated image. In the generator part, we use consecutive residual blocks with the content loss to retrieve the photo-realistic original image. It can restore the edges and enhance the resolution by ×2, ×4, and even ×8 times without seriously hampering the image quality. The model is tested with a variety of low-resolution microscopic sample images and successfully generates high-resolution directional view images with better illumination. The quantitative analysis shows that the proposed model performs better for microscopic images than the existing algorithms.

## 1. Introduction

An optical microscope is a piece of magnification equipment for viewing and observing microscopic objects. The microscope is extensively used in many different fields, such as biomedical science, nanophysics, and medical science [1,2,3]. Generally, it consists of an objective lens and a tube lens. The specimen is placed under the objective lens, and the magnified object is viewed from the tube lens also known as the eyepiece. Magnification depends on the focal length of these two lenses. However, conventional two-dimensional (2D) microscopy only enhances the resolution and provides 2D information that cannot perceive the parallax and the depth information. It is a major problem where three-dimensional (3D) information is necessary. To overcome this situation, different types of 3D microscopy such as confocal [3], stereoscopic [4], integral imaging microscopy (IIM)/light field microscopy (LFM) [2,5,6], and integral imaging holographic microscopy [7] have been proposed. Among these, IIM/LFM microscopy provides full 3D and parallax information. Jang and Javidi employed the first integral imaging technique for microscopic objects [8]. Later, Levoy et al. proposed and developed the first IIM microscopy [9]. An integral imaging system consists of a camera with an *MLA* that generates elemental images. The main advantage of this system is that it takes a single shot, whereas *MLA* works similar to multiple cameras. That is why the setup is simpler and the acquisition accuracy is far better in this case. Adjacent lens array generates perspective views in the principle of the stereoscopic imaging system. In fact, the IIM provides multiple perspective views (depends on the lens array) than the usual stereoscopic system. Since the IIM captures multiple views under a single camera, the resolution becomes small; hence, resolution enhancement is required.

Different resolution enhancement techniques for IIMs have been proposed [5,10,11,12,13,14,15,16,17]. Some of them are fully mechanical, such as synchronously moving lens arrays [13], pinhole array-based *MLA* intensity distribution methods [18], and time-multiplex systems combining low-resolution images [14]. It has a relationship between the lens array (LA) size with resolution. A larger LA enhances the resolution but reduces the depth of field (DOF). To optimize this tradeoff between them, the *MLA*-shifting method was proposed [10]. A mechanically controlled piezo-actuator was used to move the *MLA* in the vertical and horizontal directions (25 μm per step), but *MLA* shifting in the microscale is error-prone. To mitigate this problem, the interpolation-based intermediate view elemental image (IVEI) generation method was proposed using a graphic processing unit (GPU) [12]. IVEI is an interpolation-based technique where neighboring pixels are used to reconstruct another pixel. Later, the resolution was improved by applying the iterative bilinear interpolation method, which generates an orthographic view image (OVI) from the neighboring elemental image (EI) [19]. This is a relatively fast and efficient method; however, the main limitation is that the image quality dramatically decreases after some iterations. Recently, Kwon et al. proposed a deep learning-based resolution enhancement method for IIM [5] where multiple degradations based super-resolution algorithm is employed. It can enhance the resolution well; however, the quality decreases dramatically for the higher scaling factor. Hence, we focus on a scale-invariant image reconstruction system.

In this work, we improve the IIM resolution using a deep learning-based super-resolution (SR) algorithm. The resolution of conventional microscopy is limited by the lens property; however, it is possible to enhance by using resolution enhancement methods, such as classical iterative interpolation-based methods [6,12,19] and deep learning-based methods [5,15]. The quality enhancement method takes place in the OVI. Since the resolution of the OVI is small, multiple time upscaling causes a low-quality output. To solve this problem, a deep learning-based SR algorithm is applied to the directional view images that scale up 2, 4, and 8 times larger than the original image by retaining the photo-realistic natural image. Due to the characteristics (poor lighting, distortion, etc.) of IIM image, the existing network cannot be applied as it is. In this study, the network is designed considering the IIM characteristics. Unlike other super-resolution algorithms, the proposed deep learning-based algorithm performs better for IIM. First, it retrieves edges and later synthesizes the color; on the other hand, existing algorithms perform this all together. Hence, the accuracy is significant.

The rest of the paper is organized as follows. In Section 2, we describe the necessary background for a better understanding of this work. The proposed super-resolution algorithm architecture and the processes are described in Section 3. The experimental setup and the quality measurement criteria are shown in Section 4. The result is discussed in Section 5 where we compared the result with different existing algorithms. Finally, the conclusion is provided in Section 6.

## 2. Background of IIM and Super Resolution

### 2.1. Integral Imaging Microscopy

**Figure 1:**
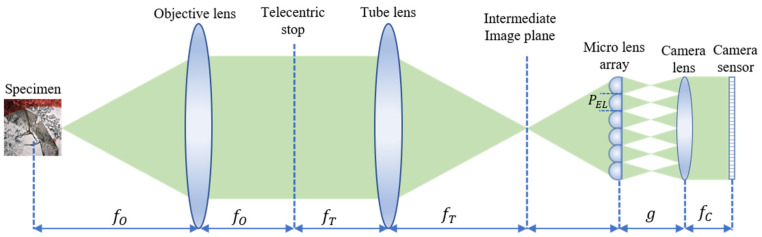
Schematic diagram of the integral imaging microscopy (IIM) capturing system. A specimen is placed in front of the objective lens and magnified through the tube lens; a micro lens array is placed in front of the sensor to capture the elemental image (*EI*).

IIM consists of a conventional microscope and an MLA. The basic schematic diagram of the IIM is shown in Figure 1. An infinity-corrected optical system is placed between the intermediate image plane and the specimen. The sensor captures the elemental image array (EIA) through *MLA* installed in front of the camera lens (CL). The OVI is reconstructed from the disparity information using the EIA. The object point (*x*, *y*, *z*) is imaged on the EIA plane through the CL and elemental lens (*EL*) as Equation (1):

(1) $$\left\{\begin{array}{c} X_{EI\left(i,j\right)}=\frac{f_{MLA}f_{C}\left(i\;\times \;P_{EL}-x\right)-f_{C}i\;\times \;P_{EL}\left(z-f_{MLA}\right)}{\left(g-f_{MLA}\right)\left(z-f_{MLA}\right)}\\ Y_{EI\left(i,j\right)}=\frac{f_{MLA}f_{C}\left(j\;\times \;P_{EL}-y\right)-f_{C}j\;\times \;P_{EL}\left(z-f_{MLA}\right)}{\left(g-f_{MLA}\right)\left(z-f_{MLA}\right)} \end{array}\right.$$

where $f_{MLA}$ and $f_{C}$ are the focal lengths of the micro lens array and camera lens, respectively. Additionally, $P_{EL}$ represents the pitch of the elemental lens, which is the distance between one lens to another. The distance between the *MLA* and the camera lens is defined as g. The lens position is denoted by *i* and *j*. However, the disparity between the camera lens and the EL should be considered as Equation (2):

(2) $$\left\{\begin{array}{c} \Delta X_{I}=\frac{f_{MLA}f_{C}P_{EL}\left(i_{2}-i_{1}\right)}{\left(g-f_{MLA}\right)\left(z-f_{MLA}\right)}\\ \Delta Y_{I}=\frac{f_{MLA}f_{C}P_{EL}\left(j_{2}-j_{1}\right)}{\left(g-f_{MLA}\right)\left(z-f_{MLA}\right)} \end{array}\right.$$

where *i* and *j* are the same as in Equation (1). This is the depth information and viewpoint obtained for each image. If the number of lenses in an *MLA* increases, the resolution is enhanced. However, there is a tradeoff between depth and resolution regarding the number of MLs. The number of directional view images depends on the resolution of EI, and the resolution of the sub-image depends on the number of EIs, as well as the ML. The generation of OVI from EIA is a little bit tricky. The first pixel of the first *EI* generates the first pixel of the first OVI, the first pixel of the second *EI* generates the second pixel of the first OVI, and so on. Similarly, the last pixel of the last *EI* generates the last pixel of the last OVI [12].

### 2.2. Deep Learning-Based Super-Resolution Algorithm

**Figure 2:**
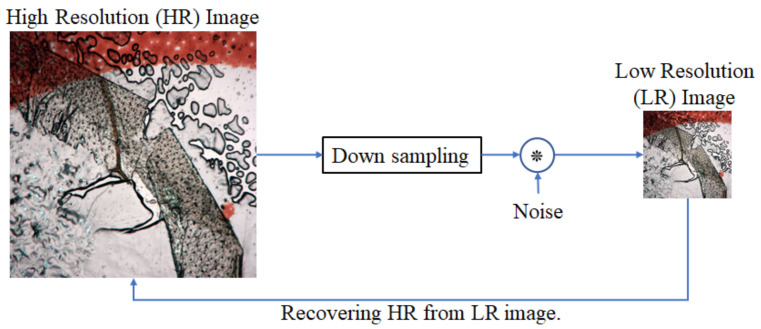
The basic concept of the single image super-resolution (SISR) algorithm. High-resolution images are downsampled to the corresponding low-resolution image and that low-resolution image inversely reconstructs the high-resolution image. The model is learned from the noise term during the training process.

Deep learning has become popular for different applications [1,20,21,22,23,24,25,26,27,28,29]; single image SR (SISR) is one of them [27,28,29,30,31]. It is a very challenging problem due to the transformation of a specific low-resolution (LR) image to a high-resolution (HR) image. The main mechanism behind SISR is that it takes the original HR image and downsample multiple times to the LR image that is fed to the network to train the model. The main working principle of the SISR algorithm is shown in Figure 2. Original HR images are downsampled and convolved with noise known as kernel by the scaling factor. The LR image *y* can be modeled as Equation (3):

(3) $$y=\left(x*n\right)↓s+N,$$

where *x* is the HR part that convolved with the noise *n*, ↓ is the downsampling operator with factor *s*. *N* (if necessary) is the independent (also known as bias) noise term. Most of the SISR algorithms work on the same concept.

There are different techniques for SISR algorithms; among them, the interpolation-based method is widely used [32,33,34]. A simple three-layer convolutional neural network (CNN)-based SR algorithm (known as SRCNN) was proposed [32]. These three nonlinear layers extract patches from the LR image map and reconstruct the HR image. Later, a pyramid structure network (LapSRN) was proposed [33]. The feature map was generated by cascading the convolutional layers; then, upscaling was performed by the cascade convolution; finally, a convolutional layer was used to predict the sub-band residuals. Recently, efficient multiple degradation-based algorithms, SRMD, was proposed [34]. It performs better in a noise-free (SRMDNF) situation. Recently, HDRN is proposed where a hierarchical dense block (HDB) is used to represent the feature module [31]. The HR image is reconstructed by the sub-pixel operation where the global fusion module is employed with HDB. However, the (first proposed in 2014 [35]) generative adversarial network (GAN)-based super-resolution algorithm is becoming popular day by day. There are different variants of GAN, such as InfoGAN [36], DCGAN [37], CycleGAN [38], and SRGAN [39]. Most of them use a rectified linear unit (ReLU) activation function [40]. It is difficult to resolve the finer texture detail for the photo-realistic natural image using general SR algorithms. However, the SRGAN can mitigate this problem using the perceptual loss function. Hence, we used a modified version of the SRGAN algorithm for resolution enhancement.

**Figure 3:**
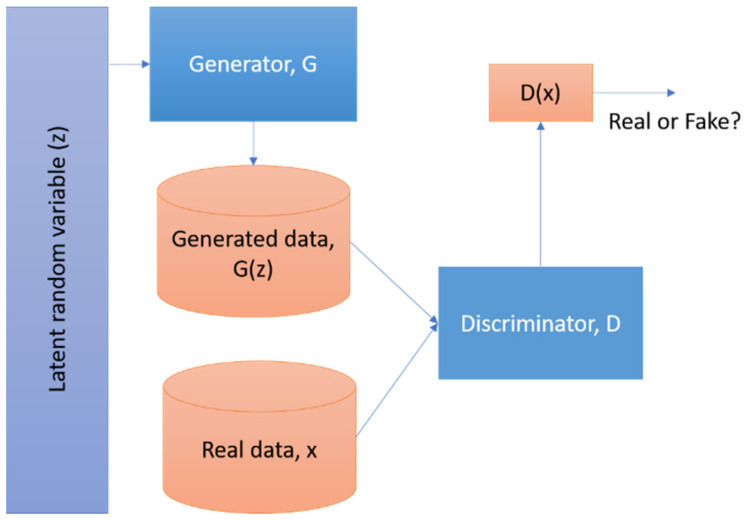
Schematic diagram of a basic Generative Adversarial Network. Latent variable or noise term is used in the generator part to generate the output, whereas the discriminator part compares the generated and original data to distinguish whether it is real or fake.

The basic concept of the GAN network is shown in Figure 3. Unlike other algorithms, it has two parts—the generator (G) and the discriminator (D) that train at the same time. The G works based on the random variable z (also known as noise). Both the generated data G(z) and the real data x are used in D to verify whether it is real or fake. It is more related to min–max rather than an optimization problem [35]. G needs to be trained simultaneously to minimize the difference between D and G. We can define it in terms of the value function V (D, G) as Equation (4):

(4) $$\underset{G}{\min}\underset{D}{\;\max}V\left(D,G\right)=\;E_{x\sim p_{data}\left(x\right)}\left[\log D\left(x\right)\right]+E_{z\sim p_{z}\left(z\right)}\left[\log(1-D(G(x)))\right]$$

Equation (4) maximizes the value function of D and minimizes G. With this method, the generator can learn to produce a high-quality similar image from the original one.

## 3. Proposed Method for IIM Super-Resolution

The whole process is divided into two major parts—IIM capture through *MLA* and resolution enhancement using the proposed GAN-based super-resolution algorithm. In the capturing process (shown in Figure 1), the specimen is placed in front of the objective lens of the microscope. The magnified image is formed in the intermediate image plane between the tube lens and the *MLA*. Each ML works as an individual image source that generates a perspective view image, combinedly known as *EI*. The *EI* cannot be directly used for resolution enhancement. Therefore, the OVI is generated from the EIA, which contains the directional view information. The resolution and the number of directional view images are the same as the number of EIs and the resolution, respectively. Each directional view image was processed through the designed algorithm; the resolution was enhanced 2, 4, and 8 times and combined again for the full visualization system.

**Figure 4:**
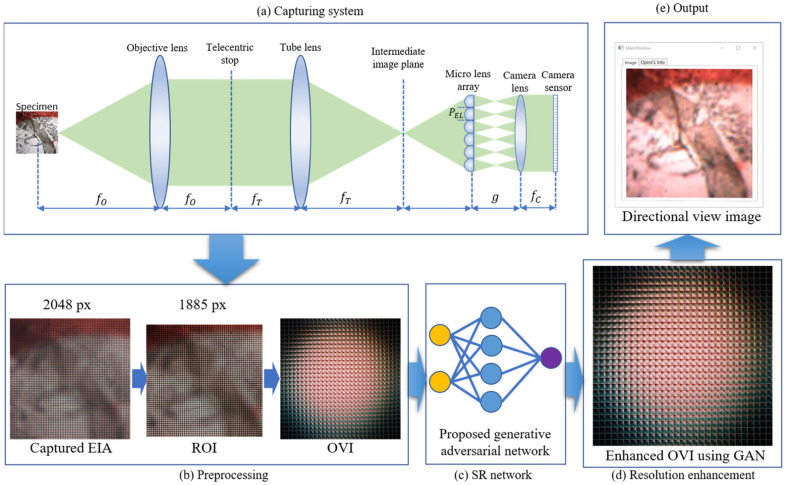
Block diagram of the proposed IIM resolution enhancement method. (**a**) An IIM capturing system where objective lens, tube lens, and micro lens array is employed to capture the EIA. (**b**) In the preprocessing part, the outer dark part is removed by cropping the elemental image array (EIA), and orthographic view image (OVI) is generated from the EIA using the pixel mapping algorithm. (**c**) A super-resolution algorithm is designed and trained using generative adversarial network (GAN). (**d**) The resolution of the OVI is enhanced using the SR network. (**e**) The resolution-enhanced directional view image is shown as an output.

**Figure 5:**
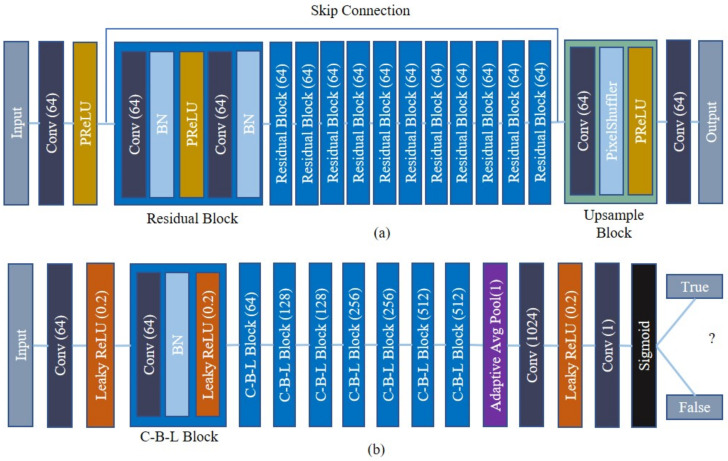
The architecture of the proposed GAN-based resolution enhancement algorithm: (**a**) generator network: the main building block of the network where noise term is used to generate the output; (**b**) discriminator network: discriminate between the generated and the real image.

Figure 4 shows the detailed block diagram of the proposed IIM resolution enhancement system. A honeybee sample (~500 μm) is taken as a specimen. The EIA is captured through a camera whose resolution is 2048 × 2048 pixels. Due to the *MLA* properties, the outer side of the captured EIA contains noise; hence, a region of interest (ROI) is selected by cropping it into 1885 × 1885 pixels. From this selected ROI, an OVI is generated using a pixel mapping algorithm [12]. In this technique, each *EI* is mapped into the corresponding view image. The OVIs are fed to the designed GAN model (Figure 5) as input. The resolution of the directional view image from the OVI is individually enhanced 2, 4, and 8 times using the proposed algorithm. The directional view image generates a perspective view, which contains parallax information providing depth perception.

The main schematic diagram of the proposed algorithm is shown in Figure 5. This network is the modified version of the SRGAN algorithm. Since the scenarios are a little different in the microscopic image than the traditional one, some modifications are performed to cope with the microscopic image. Modification and network structure are described below. As mentioned earlier, the whole network has two different sections: the generator and the discriminator.

### 3.1. The Generator Network

The LR image is taken as the input to the generator that passes through the convolution layer and parametric ReLU (PReLU) [41,42]. The PReLU can be defined as Equation (5):

(5) $$f\left(x\right)=\left\{\begin{array}{c} x_{i},\;\mathrm{if}\;x_{i}>0\\ a_{i}x_{i},\;\mathrm{if}\;x_{i}\leq 0\; \end{array}\right.$$

where $x_{i}$ is the input of the hidden layer on the *i*th channel, $a_{i}$ is the coefficient of the negative part that controls the slope. The value of $a_{i}$ is learned via backpropagation during training time. When the value of $a_{i}$ is 0, it works as a ReLU activation function. The convolution layer consists of 9 × 9 kernels and 64 feature maps with padding 4. Apart from the original SRGAN, twelve residual blocks are used in the generator network. Each block consists of two convolutions followed by batch normalization (BN); after that, a PReLU is employed; the kernel size is 3 × 3 and the padding is 1 for both convolution layers. Due to the change of parameter in each layer for training, the learning rate reduces gradually with saturating nonlinearities. In that case, BN performs normalization for each mini-batch; hence, it allows a higher learning rate [43]. In the last residual block, there are 64 channels and 64 feature maps with kernel size 3 × 3 and padding 4. To enhance the resolution of the LR input image, an upsample block is used, which consists of a convolution, pixel shuffler, and PReLU layer. A single pixel-shuffler is useful for efficient sub-pixel convolution where the super-resolution takes place in the LR instead of the HR space [44], whereas two are used in the SRGAN. It helps to retrieve the color consistency of the IIM. In this upsample block, the channel size is the same as the residual block; however, the feature map size is calculated by multiplying the channel size and the square of the up-scale factor; kernel size and the padding is 3 × 3 and 1, respectively. In the gradient-based learning method, vanishing gradient plays a vital role, sometimes the network may stop learning. To overcome this, a skip connection is established between the input and the upsampling block to prevent the gradient vanishing problem where the weight value is calculated from the previous layer. The final convolution layer contains three output channels and a 9 × 9 kernel size with padding 4.

### 3.2. The Discriminator Network

To verify the generated and original image, a discriminator network is used. After the input layer, there is a convolution (three channel and 64 feature maps) followed by a leaky ReLU layer containing a kernel size of 9 × 9 and padding 1. The leaky ReLU is almost similar to the PReLU, if the value of $a_{i}$ is fixed then it becomes leaky ReLU [45], whereas the value of $a_{i}$ is variable for PReLU. In the proposed network, the value is always set to 0.2. There are seven consecutive convolution, BN, and leaky ReLU blocks (C–B–L Block). The C–B–L block starts with 64 channels and consecutively increases by 128, 256, and 512. All C–B–L blocks have the same kernel size 3 × 3 and padding 1. We applied stride 2 only in the odd C–B–L blocks. Adaptive average polling is used before the convolution layer. Lastly, there are two convolution layers with the output channel 1024 and 1, respectively; the kernel size is 1 × 1. A leaky ReLU with a constant value of 0.2 is employed between those convolution layer. A sigmoid activation function is used to discriminate whether the output is correct or not. We use the VGG feature map (pre-trained on the ImageNet dataset) to retrieve a photo-realistic image [46], whereas perceptual loss is used in the SRGAN. During the training period, a stochastic gradient-based optimizer, Adam, is used [47]. The sample data and source code of this proposed GAN network is available on GitHub (https://github.com/shahinur-alam/IIM-GAN, accessed on 12 March 2021).

## 4. Experimental Setup and Quality Measurement Metrics

**Figure 6:**
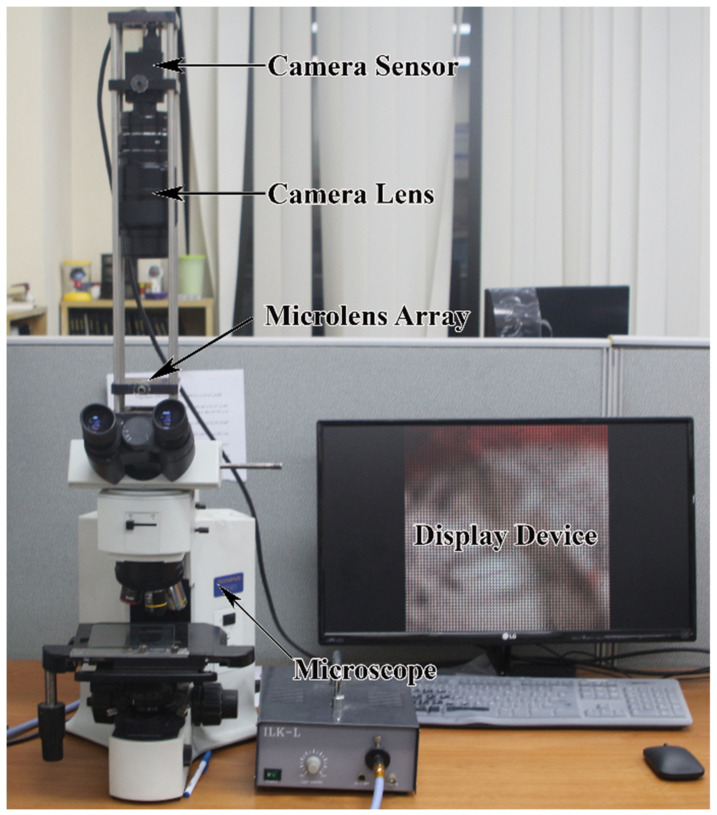
Experimental setup for the proposed IIM resolution enhancement system. The micro lens array (MLA) is placed between the camera sensor and the specimen, and the captured EIA is displayed in the display in real-time.

In the original experimental setup shown in Figure 6, we use an Olympus BX41TF microscope with 10× magnification. An *MLA* composed of 100 × 100 lenses is used for IIM; each lens diameter and focal length are 2.4 mm and 125 μm, respectively. The image is captured using the Point Grey GS3-U3-41C6C-C, 1-inch CMOS sensor through the NIKON 20 mm lens. This sensor can capture 4.1 megapixels of information at 2048 × 2048 resolution.

A high-configuration personal computer (PC) is used to train the model, which is composed of an Intel Core i7-9800X 3.80 GHz processor with 128 GB RAM. The PC operates in a Windows 10 Pro 64-bit operating system associated with NVIDIA GeForce RTX 2080 Ti GPU. We use the Python programming language in the Anaconda environment with the PyTorch library to train and test this network. Since there is a lack of IIM dataset, the network is trained using the popular Pascal VOC2012 dataset, which contains 16,700 training and 425 validation images [48]. It has twenty classes of objects, including person, animal, vehicle, furniture, etc. Compared to other existing datasets, the VOC2012 dataset is optimal in this specific application. The training time was almost 17 h. The generated model is tested and verified by the real microscopic specimens. The proposed network retrieves edges, that is why it can enhance the resolution of the microscopic image, though the VOC2012 dataset is a little different to the microscopic specimens.

There are different kinds of image quality measurement (IQM) techniques that are frequently used to compare the original and output images. Here, we employed the peak signal-to-noise ratio (PSNR), structural similarity index (SSIM), and power spectral density function (PSD).

### 4.1. PSNR

PSNR is the most commonly used IQM technique since it is simple and computationally cost-efficient [49]. It is the ratio between the maximum power and noise signal. If $I\left(x,y\right)$ is the original image and $K\left(x,y\right)$ is the generated or distorted image, then PSNR is calculated as Equation (6):

(6) $$\mathrm{PSNR}=20\log_{10}\frac{MAX^{2}\;\times \;M\;\times \;N}{\sum_{i=0}^{M-1}\sum_{j=0}^{N-1}{\left[I\left(x,y\right)-K\left(x,y\right)\right]}^{2}}$$

where *MAX* is the peak signal power. For an 8-bit general image, the *MAX* value is 255. *M* and *N* is the resolution of the image. The instantaneous pixel value is denoted by *i* and *j* for the width and height of the image, respectively. PSNR has obvious physical meaning in terms of optimization. Since it uses the mean square method, the PSNR value is always nonnegative in the decibel unit.

### 4.2. SSIM

SSIM calculates the image similarity focusing on the human visual system (HVS). Unlike PSNR, SSIM not only considers the absolute error but also focuses on structural information [50]. SSIM considers the structure (*s*), luminance (*l*), and the contrast (*c*):

(7) $$s\left(x,y\right)=\frac{\sigma_{xy}+c_{3}}{\sigma_{x}\sigma_{y}+c_{3}}$$

(8) $$l\left(x,y\right)=\frac{2\mu_{x}\mu_{y}+c_{1}}{\mu_{x}^{2}+\mu_{y}^{2}+c_{1}}$$

(9) $$c\left(x,y\right)=\frac{2\sigma_{x}\sigma_{y}+c_{2}}{\sigma_{x}^{2}+\sigma_{y}^{2}+c_{2}}$$

where $\mu_{x},\;\mu_{y},\;\sigma_{x},\;\sigma_{y}$ are the average and variance of $x_{i},\;y_{i},$ respectively. The SSIM is calculated after combining Equations (7)–(9) as:

(10) $$\mathrm{SSIM}\left(x,y\right)=\;{\left[l\left(x,y\right)\right]}^{\alpha }\;{\left[c\left(x,y\right)\right]}^{\beta }\;{\left[s\left(x,y\right)\right]}^{\gamma }$$

Equation (10) calculates the final SSIM value for two signals as well as images; α, β, and γ are positive values that represent the magnitude of those three components. Magnitude is the user-defined value.

### 4.3. PSD

The power spectral density function is one kind of no-reference image quality assessment technique [50]. The power spectrum of a signal represents the power into the frequency of that signal. The PSD is calculated using the 2D Fourier transform as Equation (11):

(11) $$\mathrm{PSD}=\log_{10}{\left|Ϝ\left[x\left(t\right)\right]\right|}^{2}$$

where *x*(*t*) is a time-series signal. However, Equation (11) provides continuous spectral information. To quantify the PSD value, a mean value is calculated from each spectral power.

## 5. Results and Discussion of the Proposed Resolution Enhancement Method

**Figure 7:**
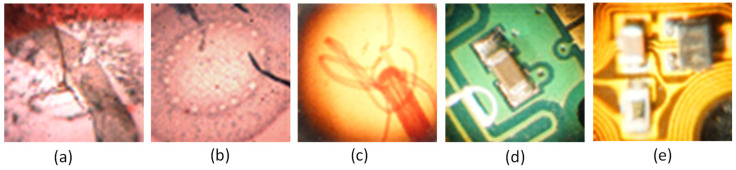
Different types of specimen: (**a**) honeybee, (**b**) Z. Mays, (**c**) hydra, (**d**) chip, and (**e**) printed circuit board (PCB).

**Figure 8:**
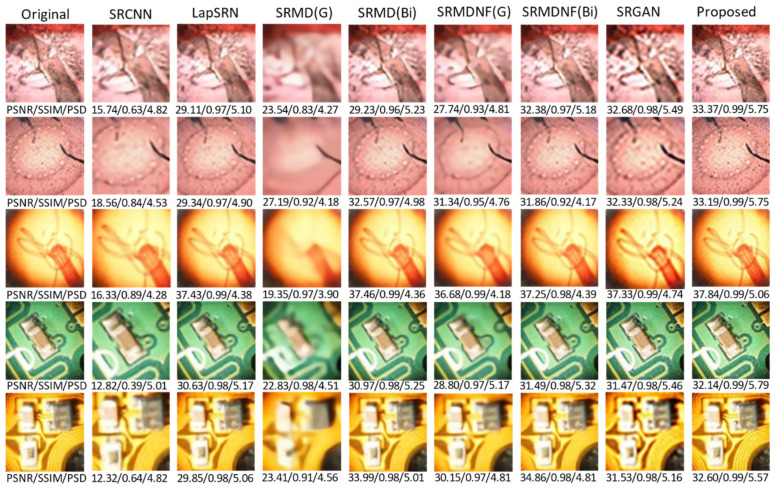
×2 upscaling comparison. The SRCNN, LapSRN, SRMD, SRMDNF, and SRGAN algorithms are compared with the proposed method. Most of the cases, the proposed method performs better than others, and the super-resolved image is almost indistinguishable from the original.

**Figure 9:**
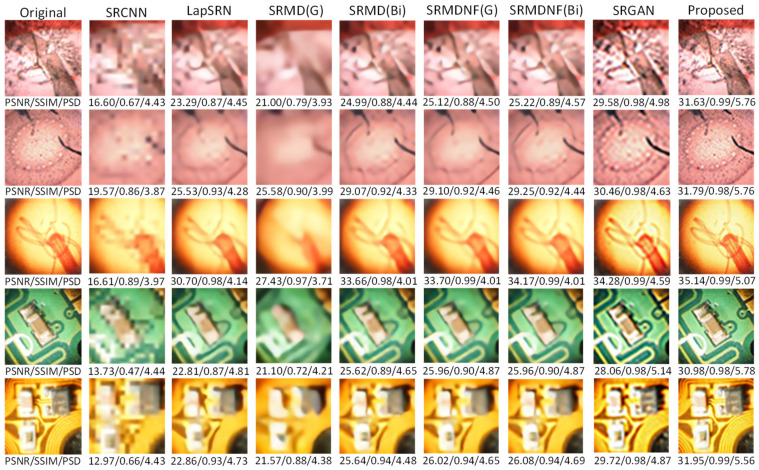
×4 upscaling comparison. The SRCNN, LapSRN, SRMD, SRMDNF, and SRGAN algorithm are compared with the proposed method. In all cases, the proposed method performs better than others, and the super-resolved image is almost indistinguishable from the original.

In this research, five different kinds of microscopic specimens (honeybee, Zea Mays (Z. Mays), hydra, chip resistor, and printed circuit board (PCB)) are used. The specimens are collected by an IIM composed of a traditional microscope and *MLA*. All specimens are shown in Figure 7. The PSNR, SSIM, and PSD are used to evaluate the enhanced image quality. The visual results are shown in Figure 8 and Figure 9 for ×2 and ×4 upscaling of different specimens, respectively. It is observed from these figures that the output of the proposed algorithm is almost similar to the original image. Only a single OVI from each specimen is displayed here for better understanding. Table 1 and Table 2 show the PSNR, SSIM, and PSD comparison for ×2 and ×4 upscaling for LapSRN [33], SRCNN [32], SRMD [34], SRMDNF [34], and SRGAN [39]. The highest obtained result is shown in boldface (Table 1 and Table 3). There are two upsampling and downsampling mechanisms for SRMD and SRMDNF, and it is shown that the PSNR, SSIM, and PSD values are higher in most of the cases for bicubic interpolation than the general method.

**Figure 10:**
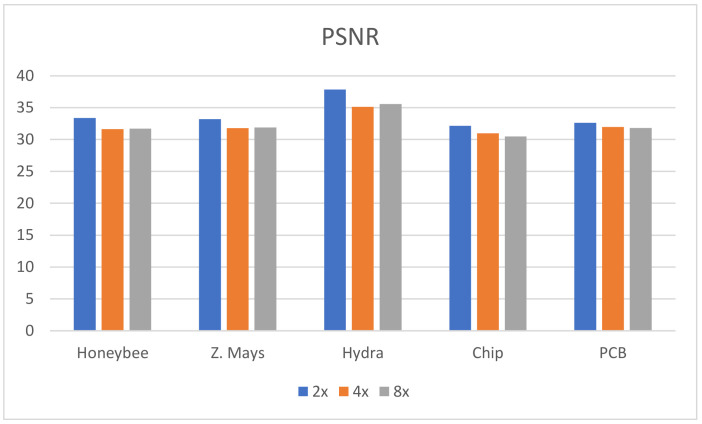
PSNR comparison between the ×2, ×4, and ×8 upscaling factors. The maximum value found for the ×2; however, compared to ×8, the difference is not more than 2.

**Figure 11:**
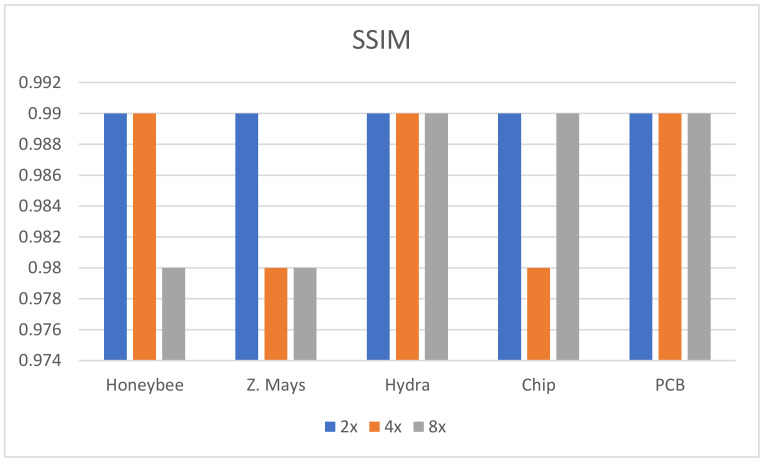
SSIM comparison between the ×2, ×4, and ×8 upscaling factors. The best result is found for ×2 upscaling, which means the output and original image is more similar.

**Figure 12:**
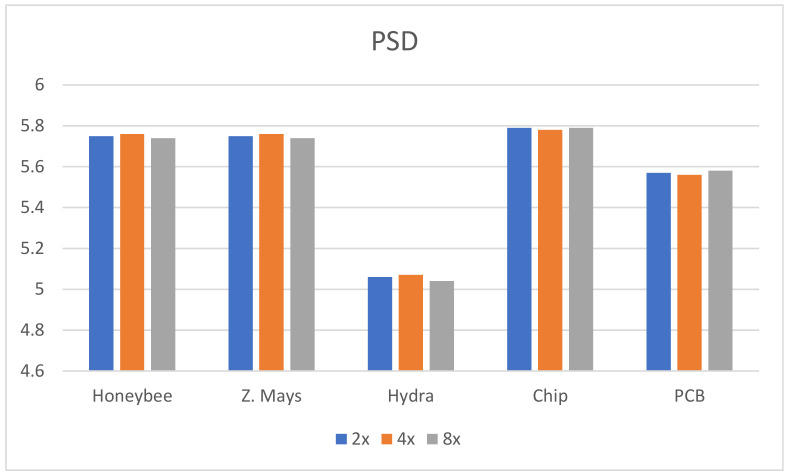
PSD comparison between the ×2, ×4, and ×8 upscaling factors. The PSD value varies across different specimen. However, in most of the cases, the ×2 upscaling performs better.

From the table, in most cases, the PSNR, SSIM, and PSD values are higher for the proposed algorithm, which verifies that the method is highly suitable for microscopic specimens. There is only one case found for the PCB ×2 upscaling factor where the PSNR value is higher for the SRMDNF bicubic interpolation technique. This is because of the algorithm properties and PSNR calculation technique. SRMDNF enhances the image quality without seriously considering the edges. If we take a closer look at Figure 7 and compare the PCB specimen with the SRMDNF and proposed algorithm, there is a huge edge difference. The microwires are clearly visible for the proposed algorithm but not in the SRMDNF (it seems like a single wire stripe). Since the PSNR considers the mean square error and the output is brighter for SRMDNF, it provides a higher PSNR value. However, the SSIM and PSD values are higher for the method applied here. It is a considerable flaw that most of the algorithm does not support (possibly very noisy and low-quality output) more than ×2 or ×4 upscaling except SRGAN, which is a great advantage for this algorithm [51]. The ×8 upscaling results for the different specimens are shown in Table 3. The results are reasonable. It is observed in Table 1, Table 2 and Table 3 that the PSNR, SSIM, and PSD values are almost identical to different quality measurement techniques, which verifies that the proposed method can retrieve a good quality image for even an ×8 upscaling factor. The comparison between the PSNR, SSIM, and PSD values for different upscaling factors are shown in Figure 10, Figure 11 and Figure 12, respectively. The quality and scaling factors are inversely proportional. The higher the scaling factor, the less image quality is. The PSNR values for honeybee, Z. Mays, hydra, chip, and PCB are (33.57, 33.19, 37.84, 32.14, 32.60), (31.63, 31.79, 35.14, 30.98, 31.95), (31.71, 31.89, 35.59, 30.48, 31.81) for ×2, ×4, and ×8 upscaling, respectively. Figure 10 shows that the PSNR value is always higher and relatively lower for the ×2 and ×8 upscaling, respectively. The structure of the generated images is also better for ×2 upscaling (shown in Figure 11), though it is very similar to others. The SSIM values for honeybee, Z. Mays, hydra, chip, and PCB are (0.99, 0.99, 0.99, 0.99, 0.99), (0.99, 0.98, 0.99, 0.98, 0.99), (0.98, 0.98, 0.99, 0.99, 0.99) for ×2, ×4, and ×8 upscaling, respectively. The PSD values are loosely related because they depend on the specimen. Brightness and sharpness are completely different from each other. The PSD values for the honeybee, Z. Mays, hydra, chip, and PCB are (5.75, 5.75, 5.06, 5.79, 5.57), (5.76, 5.76, 5.07, 5.78, 5.56), (5.74, 5.74, 5.04, 5.79, 5.18) for ×2, ×4, and ×8 upscaling, respectively. PSD values are calculated in the decibel (dB) unit. It is shown in Figure 12 that the PSD values vary across different specimens. The quantitative results show that the output image is good enough to perceive a better microscopic view. However, the adversarial network produces some additional noises.

Due to the multiple ranges of the upscaling factor, the proposed method does not require the generation of any iterative interpolation-based intermediate view image [5]; in fact, the generation time dramatically increases after each iteration. However, the proposed method reduces the calculation complexity by retaining the image quality. The proposed algorithm takes, on average, 0.025 s to generate a one-directional view image. The training and testing times are calculated using the PyTorch default function.

## 6. Conclusions

In this paper, a useful and efficient deep learning-based resolution enhancement method for IIMs is presented. The proposed adversarial-based network efficiently handles photo-realistic images and reconstructs images similar to the original one. The EIA is captured through a camera sensor attached to the lens array and a 2D microscope. Then, the OVI is generated from the EIA according to the mapping algorithm. OVI contains multiple directional view images that generate 3D perception to the observer. The directional view images are directly used to feed the SR algorithm. As a result, we can obtain a high-quality resolution enhanced image.

The quantitative analysis of the PSD, SSIM, and PSNR shows that the proposed method outperforms all state-of-the-art algorithms. This algorithm requires a very short time to generate a single-view image. Additionally, it is shown that the PSNR, SSIM, and PSD values are almost identical for ×2, ×4, and ×8 upscaling factors, which is a great advantage for the proposed system. Furthermore, it indicates that the enhanced image quality rarely depends on the scaling factor. In future work, getting better resolution-enhanced images and faster generation will be the main focus of this research. However, different noise suppression techniques will be applied in future work, and the state-of-the-art deep learning algorithms will be compared with our improved deep learning model. Another important thing is that the IIM dataset is not adequate to date. In future work, we will also focus on making datasets for the deep learning network.

## Figures and Tables

**Table 1:** PSNR, SSIM, and PSD comparison of the super resolved OVI (×2) using different algorithms. Most of the cases the proposed method performs better except the PSNR of the PCB specimen. However, the other parameters are still better.

		Honeybee	Z. Mays	Hydra	Chip	PCB
SRCNN	PSNR	15.74	18.56	16.33	12.82	12.32
	SSIM	0.63	0.84	0.89	0.39	0.64
	PSD	4.82	4.53	4.28	5.01	4.82
LapSRN	PSNR	29.11	29.34	37.43	30.63	29.85
	SSIM	0.97	0.97	0.99	0.98	0.98
	PSD	5.10	4.90	4.38	5.17	5.06
SRMD (general)	PSNR	23.54	27.19	19.35	22.83	23.41
	SSIM	0.83	0.92	0.97	0.98	0.91
	PSD	4.27	4.18	3.90	4.51	4.56
SRMD (bicubic)	PSNR	29.23	32.57	37.46	30.97	33.99
	SSIM	0.96	0.97	0.99	0.98	0.98
	PSD	5.23	4.98	4.36	5.25	5.01
SRMDNF (general)	PSNR	27.74	31.34	36.68	28.80	30.15
	SSIM	0.93	0.95	0.99	0.97	0.97
	PSD	4.81	4.76	4.18	5.17	4.81
SRMDNF (bicubic)	PSNR	32.38	31.86	37.25	31.49	**34.86**
	SSIM	0.97	0.92	0.98	0.98	0.98
	PSD	5.18	4.17	4.39	5.32	4.81
SRGAN	PSNR	32.68	32.33	37.33	31.47	31.53
	SSIM	0.98	0.98	0.99	0.98	0.98
	PSD	5.49	5.24	4.74	5.46	5.16
Proposed	PSNR	**33.37**	**33.19**	**37.84**	**32.14**	32.60
	SSIM	**0.99**	**0.99**	**0.99**	**0.99**	**0.99**
	PSD	**5.75**	**5.75**	**5.06**	**5.79**	**5.57**

**Table 2:** PSNR, SSIM, and PSD comparison of the super resolved OVI (×4) using different algorithms. The proposed algorithm performs better in all cases.

		Honeybee	Z. Mays	Hydra	Chip	PCB
SRCNN	PSNR	16.60	19.57	16.61	13.73	12.97
	SSIM	0.67	0.86	0.89	0.47	0.66
	PSD	4.43	3.87	3.97	4.44	4.43
LapSRN	PSNR	23.29	25.53	30.70	22.81	22.86
	SSIM	0.87	0.93	0.98	0.87	0.93
	PSD	4.45	4.28	4.14	4.81	4.73
SRMD (general)	PSNR	21.00	25.58	27.43	21.10	21.57
	SSIM	0.79	0.90	0.97	0.72	0.88
	PSD	3.93	3.99	3.71	4.21	4.38
SRMD (bicubic)	PSNR	24.99	29.07	33.66	25.62	25.64
	SSIM	0.88	0.92	0.98	0.89	0.94
	PSD	4.44	4.33	4.01	4.65	4.48
SRMDNF (general)	PSNR	25.12	29.10	33.70	25.96	26.02
	SSIM	0.88	0.92	0.99	0.90	0.94
	PSD	4.50	4.46	4.01	4.87	4.65
SRMDNF (bicubic)	PSNR	25.22	29.25	34.17	25.96	26.08
	SSIM	0.89	0.92	0.99	0.90	0.94
	PSD	4.57	4.44	4.01	4.87	4.69
SRGAN	PSNR	29.58	30.46	34.28	28.06	29.72
	SSIM	0.98	0.98	0.99	0.98	0.98
	PSD	4.98	4.63	4.59	5.14	4.87
Proposed	PSNR	**31.63**	**31.79**	**35.14**	**30.98**	**31.95**
	SSIM	**0.99**	**0.98**	**0.99**	**0.98**	**0.99**
	PSD	**5.76**	**5.76**	**5.07**	**5.78**	**5.56**

**Table 3:** ×8 upscaling directional view image comparison using the proposed algorithm.

	Honeybee	Z. Mays	Hydra	Chip	PCB
PSNR	31.71	31.89	35.59	30.48	31.81
SSIM	0.98	0.98	0.99	0.99	0.99
PSD	5.74	5.74	5.04	5.79	5.18

## Data Availability

Publicly available data were analyzed in this study. This data can be found here: [https://github.com/shahinur-alam/IIM-GAN, accessed on 12 March 2021].

## Abbreviations

C–B–L	Convolution, batch normalization, leaky ReLU
CL	Camera lens
DOF	Depth of field
*EI*	Elemental image
EIA	Elemental image array
EL	Elemental lens
GAN	Generative adversarial network
GPU	Graphic processing unit
HR	High resolution
HVS	Human visual system
IIM	Integral imaging microscopy
IQM	Image quality measurement
IVEI	Intermediate view elemental image
LA	Lens array
LFM	Light field microscopy
LR	Low resolution
*MLA*	Micro lens array
OVI	Orthographic view image
PCB	Printed circuit board
PSD	power spectral density
PSNR	Peak signal-to-noise ratio
ReLU	Rectified linear unit
PReLU	Parametric rectified linear unit
SISR	Single image super-resolution
SR	Super resolution
SSIM	Structure similarity index
